# The pathological and outcome characteristics of renal lesions in Crohn’s disease

**DOI:** 10.1186/s12882-022-02883-8

**Published:** 2022-07-18

**Authors:** Zhihui Yang, Xiaochang Xu, Yejing Dong, Yimin Zhang

**Affiliations:** The Division of Nephrology, The Sixth Affiliated Hospital, Sun Yat-sen University, Guangdong Provincial Key Laboratory of Colorectal and Pelvic Floor Diseases, Guangzhou, China

**Keywords:** Crohn’s disease, Renal pathology, Outcome, Proteinuria, Hematuria

## Abstract

**Background:**

The inflammatory bowel disease, containing Crohn’s disease and ulcerative colitis, was rare in the population, especially in the complication of kidney disease. A few studies had found proteinuria played a potential indicator of inflammatory bowel disease occurrence and activity. This study aimed to better define the histopathologic spectrum and study the outcome of renal disease in Crohn’s disease.

**Methods:**

A retrospective study of 3557 Crohn's disease from January 1^st^, 2016 to July 1^st^, 2021 in the Sixth Affiliated Hospital of Sun Yat-sen University identified 20 (0.56% [20/3557]) patients who underwent kidney biopsy. All biopsy specimens were examined by standard procedures containing light microscopy, immunofluorescence, and electron microscopy.

**Results:**

Twenty cases were shown in this review study. Subnephrotic proteinuria (30% [6 of 20]), persistent hematuria and proteinuria (25% [5 of 20]), and isolated hematuria with acanthocytes (25% [5 of 20]) were the main indications for kidney biopsy. The most common diagnosis was IgA nephropathy (70% [14/20]), followed by minimal change disease (10% [2/20]), acute interstitial nephritis (5% [1/20]), granulomatous interstitial nephritis (5% [1/20]), non-IgA mesangial proliferative nephritis (5% [1/20]) and thin basement membrane nephropathy (5% [1/20]). The Lee classification of IgA nephropathy was mostly II or III level. Glomerular mesangial hyperplasia was the most common pathologic manifestation according to the MEST-C Sore. After twelve-month treatment, the majority of patients turned to complete remission of renal disease by measuring proteinuria, while 3 patients still stayed in the relapse stage and 6 patients turned to partial remission by measuring hematuria.

**Conclusions:**

IgA nephropathy is the most common kidney biopsy diagnosis in Crohn's disease. Renal damage in Crohn's disease mainly involves the glomerulus, especially the mesangial matrix. After the treatment, proteinuria might be in remission, but hematuria remains.

## Background

Inflammatory bowel disease (IBD) is a chronic and recurrent inflammatory gastrointestinal tract disease, including Crohn's disease (CD) and ulcerative colitis. Its etiology and pathogenesis are unclear. It may be related to the environment, genetic composition, gut microbiota, and immune response [1]. A retrospective study of patients with inflammatory bowel disease in China found that the crude prevalence of CD in mainland China was 0.46/100,000 [2]. About 4%-23% of IBD patients developed urinary complications, such as kidney stones, renal amyloidosis, glomerulonephritis, and renal tubulointerstitial disease [3, 4]. In addition to the low incidence, the types of CD-associated renal pathology were not identical in American, Finland, or Egyptian populations [5–7]. Proteinuria was an indicator of renal damage. It had an obvious association with the development and the activity of CD [8–11], but the relationship between proteinuria and the outcome of CD-associated patients was absent. The purpose of this study is to evaluate renal pathological features in 20 Chinese patients and the outcome of renal disease by measuring proteinuria.

## Methods

### Patient population

**Fig. 1:**
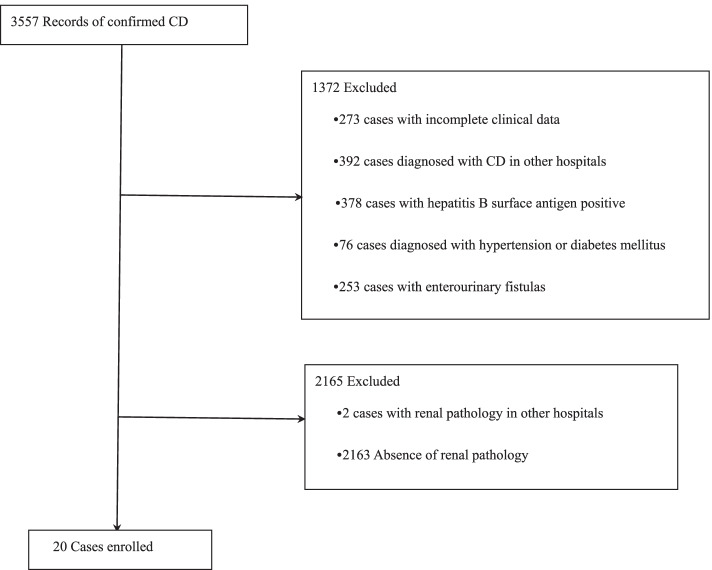
Study flow diagram that showed the process of enrollment and exclusion

We reviewed all 3557 Chinese patients who were diagnosed with CD from January 1^st^, 2016 to July 1^st^, 2021 in the Sixth Affiliated Hospital of Sun Yat-sen University. All patients were diagnosed with CD by World Gastroenterology Organization Global Guidelines [12]. We identified 20 patients who fulfilled the criteria: (1) conformity to diagnostic criteria of CD, (2) renal parenchymal damage and undergoing kidney biopsy in our hospital, (3) no current medication for renal disease, (4) excluding patients with hypertension, diabetic mellitus, hepatitis B surface antigen positive and enterourinary fistulas (Fig. 1). The demographic, clinical characteristics, and renal pathology of the 20 patients were collected before renal treatment in this study. We compared the change of proteinuria and hematuria in urinalysis measuring by dry chemistry method and analyzed the activity of CD after twelve-month treatment. We gained clinical information approved by the Ethics Committee of the Sixth Affiliated Hospital of Sun Yat-sen University (ID: 2021ZSLYEC-421).

### Study definitions

The estimated glomerular filtration rate (eGFR) was calculated using the Chronic Kidney Disease Epidemiology Collaboration Formula (CKD-EPI). Crohn’s disease activity index (CDAI) was based on the CDAI calculation method of Best [13]. The active CD was defined as CDAI ≥ 150 and quiescent disease was defined as CDAI < 150 [13]. Duration of CD referred to the time interval from the first symptom of CD to data collection. Antibiotics referred to the use of cephalosporin, sulfonamide, or anti-anaerobic bacteria drugs.

The outcome of proteinuria or hematuria: complete remission (CR) was defined as urinalysis change from negative, trace or ≥ 1 + to negative or trace. Partial remission (PR) was considered to be present if urinalysis decreased more than one stage but remained ≥ 1 + . Relapse (R) was defined as urinalysis change from negative or trace to ≥ 1 + or increased more than one stage but remained ≥ 1 + .

The outcome of CD: remission was defined as CDAI change from ≥ 150 or < 150 to < 150. Response was considered to be present if CDAI decreased by 70 or more but remained ≥ 150. Relapse was defined as CDAI change from < 150 to ≥ 150 or increased by 70 or more but remained ≥ 150 [14].

### Kidney biopsy

Kidney biopsy indications: nephrotic-range proteinuria (10% [2 of 20]), subnephrotic proteinuria (30% [6 of 20]), persistent hematuria and proteinuria (25% [5 of 20]), isolated hematuria with acanthocytes (25% [5 of 20]), and impaired kidney function (serum creatinine > 1.5 mg/dl) (10% [2 of 20]) were indications for renal biopsy [6] (Table 2).

All renal biopsy native specimens were operated by an experienced renal specialist team in the department of pathology according to a standardized process [15].

**Fig. 2:**
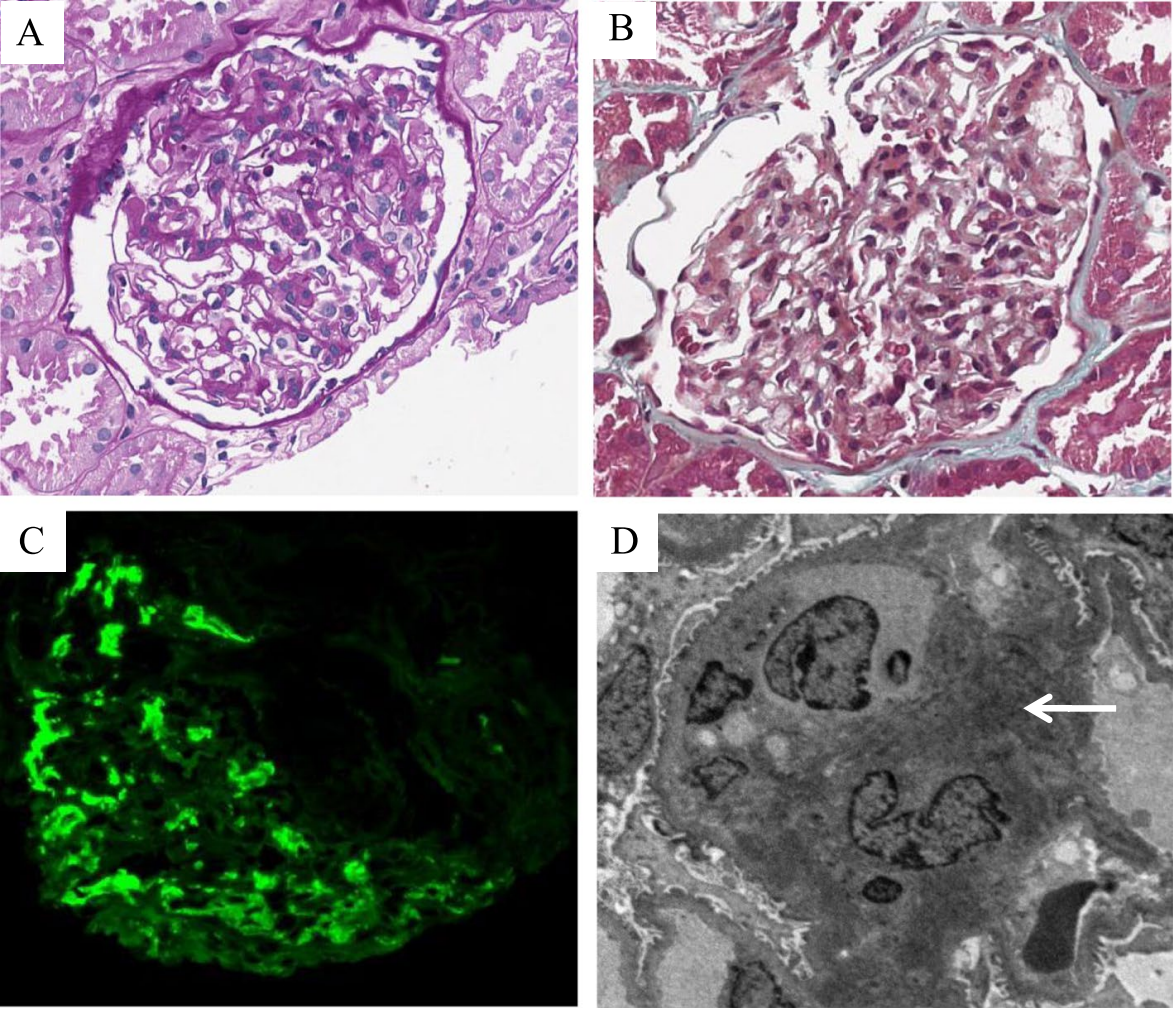
Pathologic features of IgA nephropathy associated with Crohn’s disease. **A** Glomeruli with mild segmental mesangial matrix expansion and mesangial hypercellularity (Periodic acid-Schiff, original magnification × 400). **B** Glomeruli with segmental mesangial matrix expansion, mesangial hypercellularity, and the not thickened basement membrane (Masson, original magnification × 400). **C** Glomeruli with massive IgA deposits in the mesangial region (Immunofluorescence, original magnification × 400). **D** Electron-dense deposits in the mesangial region (Arrow) (Electron microscopy, original magnification × 3000)

**Fig. 3:**
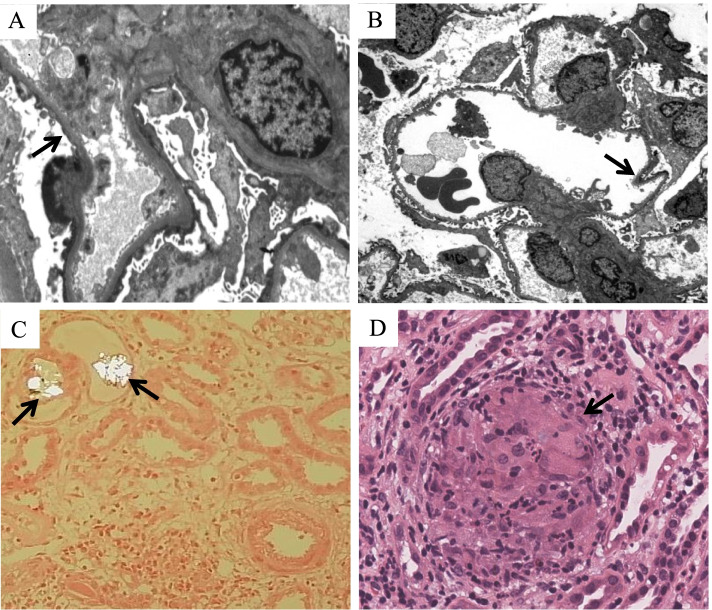
Pathologic features of minimal change disease, thin-basement-membrane nephropathy, and interstitial nephritis associated with Crohn’s disease. **A** Minimal change disease with the diffuse fusion of foot processes (Arrow) (Electron microscopy, original magnification × 4000). **B** Thin-basement-membrane nephropathy with the basement membrane less than 250 nm and segmentally shriveled (Arrow) (Electron microscopy, original magnification × 800). **C** Acute interstitial nephritis with predominantly lymphocytes and the irregular oxalate crystals deposited in the lumen of the renal tubules under polarized light (Arrows) (Hematoxylin and eosin, original magnification × 200). **D** Granulomatous interstitial nephritis with several noncaseating granulomas in the renal interstitium (Arrow) (Hematoxylin and eosin, original magnification × 400)

Specimen were treated with HE (hematoxylin and eosin), PAS (periodic acid-Schiff reagent), PASM (periodic acid-silver methenamine), and Masson trichrome. All samples were taken with immunofluorescence and electron microscopy (Fig. 2) (Fig. 3).

### Statistical analyses

The collected clinical data were analyzed by SPSS25.0 statistical software. The quantitative variables were expressed as mean±standard deviation (SD). The qualitative variables were expressed as frequencies (percentage).

## Results

### Clinical and laboratory characteristics

The base-line clinical data for 20 Chinese patients with CD at the onset of renal disease were shown in Table 1. The data were summarized and the results were shown in Table 2 and Table 3. There were 11 males and 9 females with the mean (± SD) body mass index of 19.93±2.84 kg per m^2^. The age distribution at kidney biopsy was mainly between 20 and 40 years old. The age at kidney biopsy of patients was mainly older than the age at CD diagnosis. However, one patient developed kidney disease followed by the confirmed CD (Table 1). The mean (± SD) urinary protein excretion was 0.84±1.25 g per 24 h. Excreting less than 2 g of protein per 24 h was mainly characteristic of patients. Hematuria occurred in 85% of patients. Subnephrotic proteinuria was the majority of indications for kidney biopsy. One patient [(5% (1/20)] developed acute kidney injury and 14 patients [(70% (14/20)] developed chronic kidney disease. ANCA tests were negative in all patients. Two patients diagnosed with IgA nephropathy had nephrolithiasis. Patients with active CD were 65%. Twelve months was the duration of follow-up after treatment.

**Table 1:** Demographic and outcome of 20 patients with Crohn’s disease referred for kidney biopsy after twelve-month treatments in detail

Patient	Sex	Age at kidney biopsy (yr)	Age at CD diagnosis (yr)	Renal pathology	CD treatment	Renal treatment	Outcome of CD	Outcome of proteinuria	Outcome of hematuria
1	M	45	43	IgA nephropathy	Thalidomide	RAS inhibitors, steroids intravenously	Remission	CR	CR
2	M	37	36	IgA nephropathy	Thalidomide	-	ND	ND	ND
3	F	17	15	IgA nephropathy	Methylprednisolone tablets, methotrexate	-	Remission	CR	PR
4	M	36	22	IgA nephropathy	Infliximab, azathioprine	-	Remission	CR	R
5	M	31	21	IgA nephropathy	Azathioprine, enteral nutrition	-	Remission	CR	CR
6	F	28	27	IgA nephropathy	Infliximab	Pozzi regimen	ND	ND	ND
7	F	16	18	Minimal change disease	Methylprednisolone tablets and azathioprine	Steroids intravenously	Remission	CR	CR
8	M	34	26	Minimal change disease	Enteral nutrition	RAS inhibitors	Response	CR	PR
9	F	19	18	Thin-basement-membrane nephropathy	Infliximab, azathioprine	-	Remission	CR	PR
10	F	38	37	IgA nephropathy	Thalidomide, enteral nutrition	-	Remission	CR	R
11	F	52	51	Acute interstitial nephritis	Thalidomide	Steroids intravenously	Remission	CR	CR
12	M	23	22	non-IgA mesangial proliferative nephritis	Infliximab, azathioprine	-	Response	CR	R
13	F	25	24	IgA nephropathy	Infliximab	Pozzi regimen	Remission	CR	CR
14	M	68	55	IgA nephropathy	Enteral nutrition	Prednisone acetate tablets, mycophenolate mofetil	Remission	R	PR
15	M	34	32	IgA nephropathy	Thalidomide	RAS inhibitors	Relapse	CR	CR
16	M	20	17	IgA nephropathy	Infliximab, azathioprine	Pozzi regimen	Remission	R	PR
17	F	65	42	Granulomatous interstitial nephritis	Infliximab	Prednisone acetate tablets	Remission	CR	CR
18	F	31	24	IgA nephropathy	Mesalamine, azathioprine	RAS inhibitors	ND	ND	ND
19	M	24	19	IgA nephropathy	Infliximab, azathioprine	RAS inhibitors	Remission	CR	PR
20	M	27	25	IgA nephropathy	Infliximab, azathioprine	-	Remission	CR	CR

*M* Male, *F* Female, *ND* No data. “-” referred to without any treatments

Outcome of CD: remission was defined as CDAI change from ≥ 150 or < 150 to < 150. Response was considered to be present if CDAI decreased by 70 or more but remained ≥ 150. Relapse was defined as CDAI change from < 150 to ≥ 150 or increased by 70 or more but remained ≥ 150

Outcome of proteinuria or hematuria: complete remission (CR) was defined as urinalysis change from negative, trace or ≥ 1 + to negative or trace. Partial remission (PR) was considered to be present if urinalysis decreased more than one stage but remained ≥ 1 + . Relapse (R) was defined as urinalysis change from negative or trace to ≥ 1 + or increased more than one stage but remained ≥ 1 +

**Table 2:** Demographic and renal characteristics of 20 patients with Crohn’s disease referred for kidney biopsy

Characteristic	Data
Patients — no	20
Male sex — no. (%)	11 (55%)
Age at kidney biopsy — yr	33.50±14.56
Age distribution at kidney biopsy (yr) — no. (%)	
< 20	3 (15%)
20–30	6 (30%)
30–40	7 (35%)
40–50	1 (5%)
50–60	1 (5%)
≥ 60	2 (10%)
Body mass index — Kg/m^2^	19.93±2.84
Systolic blood pressure — mmHg	110.60±13.17
Diastolic blood pressure — mmHg	71.50±7.16
Serum albumin — g per L	37.32±6.65
Serum creatinine — umol per L	115.589±113.71
Blood urea nitrogen — mmol per L	6.38±4.40
eGFR^a^ — ml per min per 1.73m^2^	99.77±44.30
eGFR^a^ category — no. (%)	
≥ 90 ml per min per 1.73m^2^	14 (70%)
60–89.9 ml per min per 1.73m^2^	3 (15%)
< 60 ml per min per 1.73m^2^	3 (15%)
Urine PH	6.28±0.82
Urinary protein excretion — g per 24 h	0.84±1.25
Degree of proteinuria (g per 24 h) — no. (%)	
≤ 0.2	7 (35%)
> 0.2 to < 2.0	10 (50%)
≥ 2.0 to < 3.5	1 (5%)
≥ 3.5 to < 5.0	2 (10%)
≥ 5.0 to 10.0	0 (0%)
≥ 10.0	0 (0%)
Hematuria — no. (%)	17 (85%)
Nephrolithiasis — no. (%)	2 (10%)
Indication for kidney biopsy — no. (%)	
Nephrotic-range proteinuria	2 (10%)
Subnephrotic proteinuria	6 (30%)
Persistent hematuria and proteinuria	5 (25%)
Isolated hematuria with acanthocytes	5 (25%)
Impaired kidney function (serum creatinine > 1.5 mg/dl)	2 (10%)
RAS inhibitors — no. (%)	5 (25%)
Steroids to treat renal disease — no. (%)	7 (35%)
Steroids intravenously — no. (%)	6 (30%)
Steroids combined with immunosuppressive agents to treat renal disease — no. (%)	1 (5%)

^a^ The estimated glomerular filtration rate (eGFR) was calculated using the Chronic Kidney Disease Epidemiology Collaboration Formula (CKD-EPI)

**Table 3:** CD-associated characteristics of the 20 patients

Clinical characteristic	Data
Montreal classification — no. (%)	
Age at CD diagnosis (A)	
A1 (Not more than 16 years)	1 (5%)
A2 (More than17 years but no more than 40 years)	15 (75%)
A3 (More than 40 years)	4 (20%)
Location (L)	
L1 (Ileal)	1 (5%)
L2 (Colonic)	0 (0%)
L3 (Ileocolonic)	19 (95%)
L4 (Upper gastrointestinal)	11 (55%)
Behavior (B)	
B1 (Non-stricturing and non-penetrating)	12 (60%)
B2 (Stricturing)	0 (0%)
B3 (Penetrating)	8 (40%)
P (Perianal disease)	14 (70%)
Age at CD diagnosis — yr	28.70±11.63
Duration of CD^a^ — mth	84.00±77.81
CDAI^b^	244.05±130.76
Active CD^c^ — no. (%)	13 (65%)
Erythrocyte sedimentation rate — mm per hr	46.70±39.59
C-reactive protein — mg per L	18.85±24.41
CD-related gastrointestinal surgery — no. (%)	7 (35%)
CD treatment — no. (%)	
5-aminosalicylic acid	1 (5%)
Glucocorticoids	2 (10%)
Biological agents	9 (45%)
Immunosuppressive agents	15 (75%)
Antibiotics^d^	0 (0%)
Enteral nutrition	4 (20%)

^a^ Duration of CD referred to the time interval from the first symptom of CD to data collection

^b^ Crohn disease activity index (CDAI) was based on the CDAI calculation method of Best

^c^ Active CD was defined as CDAI ≥ 150

^d^ Antibiotics referred to the use of cephalosporin, sulfonamide, or anti-anaerobic bacteria drugs

### Kidney biopsy findings

The most common diagnosis was IgA nephropathy (70% [14/20]), followed by minimal change disease (10% [2/20]), acute interstitial nephritis (5% [1/20]), granulomatous interstitial nephritis (5% [1/20]), non-IgA mesangial proliferative nephritis (5% [1/20]) and thin basement membrane nephropathy (TBMN) (5% [1/20]) (Table 4). The Lee classification of IgA nephropathy was mostly II or III level. Glomerular mesangial hyperplasia was the most common pathologic manifestation. IgA (intensity +  ~  +  + +), IgM (intensity +) and C3 (intensity +  ~  +  + +) staining were positive in most patients. 4 cases were positive for kappa light chains staining (intensity +) and lambda light chains staining (intensity +). One case was positive for lambda light chains staining (intensity +) and negative for kappa light chains staining. The modes of deposition were mainly diffuse massive deposits in the glomerular mesangial area (Table 6). Oxalate crystals existed in renal tubules in two cases of interstitial nephritis without nephrolithiasis. Proteinuria appeared in five patients diagnosed with IgA nephropathy, of whom two contained crescents with negative ANCA tests and one with Lee IV grade.

**Table 4:** Primary kidney biopsy findings and renal outcome from the diagnosis of renal disease to six-month and twelve-month treatment by urinalysis^a^ in 20 patients with Crohn’s disease

		Six-month follow-up		Twelve-month follow-up	
Diagnosis	Patients (n/N)	Outcome of proteinuria	Outcome of hematuria	Outcome of proteinuria	Outcome of hematuria
IgA nephropathy	14/20	10 (CR^b^), 2 (PR^c^), and two missing data	3 (CR^b^), 4 (PR^c^), 5 (R^d^), and two missing data	9 (CR^b^), 2 (R^d^), and three missing data	5 (CR^b^), 4 (PR^c^), 2 (R^d^), and three missing data
Minimal change disease	2/20	2 (CR^b^)	1 (CR^b^) and 1 (R^d^)	2 (CR^b^)	1 (CR^b^) and 1 (PR^c^)
Acute interstitial nephritis	1/20	1 (CR^b^)	1 (CR^b^)	1 (CR^b^)	1 (CR^b^)
Granulomatous interstitial nephritis	1/20	1 (CR^b^)	1 (CR^b^)	1 (CR^b^)	1 (CR^b^)
non-IgA mesangial proliferative nephritis	1/20	1 (CR^b^)	1 (R^d^)	1 (CR^b^)	1 (R^d^)
Thin-basement-membrane nephropathy	1/20	1 (CR^b^)	1 (R^d^)	1 (CR^b^)	1 (PR^c^)

^a^ Two and three patients were excluded due to a lack of follow-up data at six months and twelve months respectively

^b^ Complete remission (CR) was defined as urinalysis change from negative, trace or ≥ 1 + to negative or trace

^c^ Partial remission (PR) was considered to be present if urinalysis decreased more than one stage but remained ≥ 1 +

^d^ Relapse (R) was defined as urinalysis change from negative or trace to ≥ 1 + or increased more than one stage but remained ≥ 1 +

### Post-treatment condition

In gastrointestinal treatment, most patients [75% (15/20)] were treated with immunosuppressive agents. Several patients were treated with biological agents or enteral nutrition (Table 3). Seven patients underwent bowel resection and two patients underwent anal fistula incision or removal. Eight patients [40% (8/20)] were treated with gastrointestinal treatment without renal treatment. In renal treatment, the majority of patients were treated with glucocorticoid treatment. One of the glucocorticoid treatments was the Pozzi regimen containing an intravenous infusion of 500-1000 mg methylprednisolone once a day for three consecutive days, three times at 2-month intervals, and oral administration of 0.5 mg/kg prednisone acetate tablets once a day in six months [16]. One patient took 50 mg prednisone acetate tablets once a day combined with 0.5 g mycophenolate mofetil tablets twice a day. After twelve-month treatment, three patients were excluded due to a lack of follow-up data. 15 patients [88.2% (15/17)] turned to complete remission and 2 patients [11.8% (2/17)] translated to relapse of renal disease by observing the change of proteinuria. 3 patients [17.6% (3/17)] still stayed in the relapse stage and 6 patients [35.3% (6/17)] turned to partial remission by measuring hematuria (Table 4). The majority of patients [(82.4% (14/17)] turned to remission in the outcome of CD (Table 5).

**Table 5:** The outcome of Crohn’s disease from the diagnosis of renal disease to six-month and twelve-month treatment in 20 patients with Crohn’s disease

		Six-month follow-up^a^			Twelve-month follow-up^a^		
Diagnosis	Patients (n/N)	Remission^b^	Response^c^	Relapse^d^	Remission^b^	Response^c^	Relapse^d^
IgA nephropathy	14/20	9	2	1	10	0	1
Minimal change disease	2/20	2	0	0	1	1	0
Acute interstitial nephritis	1/20	1	0	0	1	0	0
Granulomatous interstitial nephritis	1/20	1	0	0	1	0	0
non-IgA mesangial proliferative nephritis	1/20	0	0	1	0	1	0
Thin-basement-membrane nephropathy	1/20	1	0	0	1	0	0

^a^ Two and three patients were excluded due to a lack of follow-up data at six months and twelve months respectively

^b^ Remission was defined as CDAI change from ≥ 150 or < 150 to < 150

^c^ Response was considered to be present if CDAI decreased by 70 or more but remained ≥ 150

^d^ Relapse was defined as CDAI change from < 150 to ≥ 150 or increased by 70 or more but remained ≥ 150

## Discussion

Since Hubert et al. first reported CD combined with IgA nephropathy in 1984 [17], CD combined with other renal pathological types successively appeared, such as tubulointerstitial nephritis, microvascular disease, secondary amyloidosis, minimal change disease, membranous nephropathy, etc. [4–6]. Therefore, CD was not only a chronic non-specific inflammatory gastrointestinal disease, but also affected the extra-intestinal organ, such as the kidney [4]. However, the types of renal pathology reported varied widely in different countries or regions. Ambruzs et al. [5] reported that IgA nephropathy (24% [20/83]) was the most common pathological type in 83 IBD cases in the United States. Pohjonen et al. [7] reported that among 35 IBD patients in Finland, tubulointerstitial nephritis (28.6% [10/35]) was the most common type, followed by IgA nephropathy (20% [7/35]). Elaziz et al. [6] reported that amyloidosis (25.7% [56/218]) was the most common renal pathological diagnosis in 218 Egyptian IBD patients, followed by IgA nephropathy (16.1% [35/218]). Among the 57 Egyptian patients with CD, 13 patients (22.8% [13/57]) were diagnosed with IgA nephropathy. In our study, the proportion of IgA nephropathy was significantly larger than those in Egypt, which might be related to the high incidence of IgA nephropathy in East Asia [18].

In our research, the most common diagnosis was IgA nephropathy (70% [14/20]) (Fig. 2), followed by minimal change disease (10% [2/20]), acute interstitial nephritis (5% [1/20]), granulomatous interstitial nephritis (5% [1/20]), non-IgA mesangial proliferative nephritis (5% [1/20]), and TBMN (5% [1/20]) (Fig. 3). The Lee classification of IgA nephropathy was mostly II or III level. Glomerular mesangial hyperplasia was the most common pathologic manifestation (Table 6). IgA, IgM, C3, kappa, and lambda light chains staining were positive in our patients, which was similar to primary IgA nephropathy [19]. The lambda light chains were usually brighter intensity than kappa light chains in primary IgA nephropathy [19, 20]. However, in our study, 4 cases showed equal staining for lambda and kappa light chains, which might be the difference between the primary IgA nephropathy and CD-associated IgA nephropathy. It was similar to equal staining for lambda and kappa light chains in Henoch–Schönlein purpura nephritis compared to primary IgA nephropathy [20]. Hemminger et al. reported that the intensity of lambda and kappa light chains were equal in five out of nine patients diagnosed with acute glomerulonephritis with large confluent IgA-dominant deposits secondary to liver cirrhosis [21]. Similarly, the staining for kappa light chains was equivalent or more intense than that for lambda light chains in IgA-dominant infection-associated glomerulonephritis compared to primary IgA nephropathy [22, 23]. According to our data, they appeared in men more often than women. Most patients had the active disease in CD. The majority of patients had hematuria, but the urinary protein excretion was less than 2 g/d. Compared with previous reports [5–7], the gender distribution was similar, but the patients in our data had better eGFR preservation, which might be related to the younger age distribution.

**Table 6:** The Lee classification, Oxford typing, and immunofluorescence test of 14 patients diagnosed with IgA nephropathy

Characteristic	Patients (n/N)
Lee classification of IgA nephropathy	
Grade I	1/14
Grade II	6/14
Grade III	6/14
Grade IV	1/14
Oxford typing of IgA nephropathy	
M (Glomerular mesangial hyperplasia)	11/14
E (Capillary hyperplasia)	1/14
S (Segmental glomerulosclerosis)	3/14
T (Tubular atrophy or interstitial fibrosis)	0/14
C (Crescent)	2/14
C0	0/14
C1	2/14
C2	0/14
Immunofluorescence	
IgG	0/14
IgA	14/14
IgM	11/14
C3	10/14
C1q	0/14
Fibrinogen	0/10^a^
Kappa	4/10^a^
Lambda	5/10^a^
IgG1	0/11^b^
IgG2	0/11^b^
IgG3	0/11^b^
IgG4	0/11^b^
PLA2R	0/11^b^

^a^ Four patients were excluded due to missing data on fibrinogen, kappa, and lambda

^b^ Three patients were excluded due to missing data on IgG1, IgG2, IgG3, IgG4, and PLA2R

Hubert et al. reported that one patient with CD complicated by IgA nephropathy, underwent repeated renal biopsy after control of intestinal disease symptoms. The result showed that hematuria, mesangial proliferation, and IgA deposits disappeared in that patient [17]. Forshaw et al. reported a case of CD combined with IgA nephropathy. Gastrointestinal symptoms and abnormalities in urinalysis were relieved after the surgical resection of the terminal ileum and cecum. During the follow-up, gastrointestinal symptoms, renal function, and urinalysis continued to show normal [24]. It was confirmed that the intestinal mucosa, especially the terminal ileum mucosa, was closely associated with IgA nephropathy in patients with CD. It was a similar mechanism in previous studies finding Peyer's patches at the end of the ileum causing proteinuria and renal function damage [25–27]. Based on this mechanism, the targeted release preparation of budesonide was designed by improving TARGIT starch capsule technology, which locally released active compounds in the distal ileum and the proximal colon. The systemic side effects of oral corticosteroids could be minimized, but the curative effect was achieved by reducing the level of proteinuria and improving renal function [26].

In addition to intestinal mucosal inflammation, heredity, microbes, and dietary associations were also related to IgA nephropathy. Genome-wide Association Study (GWAS) found that susceptibility genes of CARD9 and HORMAD2 for IgA nephropathy were also associated with IBD [28]. Yuan et al.found that the gene of CXCL2 was crucially associated with the immune infiltration between CD and IgA nephropathy [29]. Alteration of the microbiota could lead to ecological dysregulation, which regulated immune-mediated IBD and IgA nephropathy [30, 31]. In a study, a reduction in circulating IgA complexes and proteinuria was observed in patients with IgA nephropathy on a gluten-free diet for 6 months to 4 years [32].

The incidence of other pathological types of CD with renal damage was relatively low. Its pathogenesis remained unclear. It was previously thought that 5-aminosalicylic acid (5-ASA) might be the cause of minimal change disease, and acute and chronic interstitial nephritis [5, 33]. Ambruzs et al. reported 4 cases of CD with minimal change disease (5% [4/83]), 3 cases of which were currently taking 5-ASA [5]. Firwana et al. reported a case of CD with minimal change disease with significant improvement in renal function after 5-ASA withdrawal [34]. Then, 5-ASA-associated interstitial nephritis has received extensive attention [5, 33, 35]. It may be related to a hypersensitivity reaction, but not dose-related [33]. In our study, one patient with minimal change disease received 5-ASA, while the other did not. Patients with acute interstitial nephritis had never used 5-ASA, suggesting that minimal change disease and interstitial nephritis might be the extra-intestinal manifestations of CD, rather than the effect of 5-ASA. It was controversial whether granulomatous interstitial nephritis was caused by 5-ASA exposure or the extra-intestinal manifestations of CD [5, 36]. One patient in this group was diagnosed with granulomatous interstitial nephritis after excluding tuberculosis, sarcoidosis, granulomatosis with polyangiitis, and recent 5-ASA treatment. Epithelioid granuloma is a typical pathological change of intestinal lesions in CD [37], suggesting that CD itself can also cause interstitial nephritis.

Hyperoxaluria in CD is due to increased intestinal absorption and may lead to severe chronic interstitial nephritis and early end-stage renal disease [38]. It was noteworthy that oxalate crystals were seen in the renal tubules in both acute and granulomatous interstitial nephritis in our study, providing further evidence that CD itself, not 5-ASA, caused interstitial nephritis. In this study, 17 patients (85%) developed hematuria, most of which were IgA nephropathy, and a few were non-IgA mesangial proliferative nephritis and TBMN. These rare pathological types have been reported in a few cases [5, 7, 39]. TBMN is an inherited disease. It is characterized by hematuria, proteinuria, normal blood pressure, normal renal function, and a thinned glomerular basement membrane. TBMN inheritance is autosomal dominant and usually represents the carrier status of autosomal recessive in Alport syndrome. X-linked heredity in Alport syndrome should be excluded [40]. In this study, the patient had no family history of kidney disease, normal hearing, and vision tests, and no associated pathogenic genes were found in genetic tests. Although McCallum et al. [39] and Ambruzs et al. [5] have reported a few cases of CD combined with TBMN in the past, there was no reasonable explanation for the relationship between them, which needed further study.

In our study, proteinuria was all shown in some patients with Lee grade IV (7.14%　[1/14]) and crescent (14.28% [2/14]), revealing that the occurrence of proteinuria might indicate a severe pathological type. After twelve-month treatment, the majority of patients (82.4% [14/17]) turned to remission in the outcome of CD. 15 patients (88.2% [15/17]) turned to complete remission and 2 patients (11.8% [2/17]) translated to relapse of renal disease by observing the change of proteinuria. However, 3 patients (17.6% [3/17]) stayed in the relapse stage by measuring hematuria and 9 patients (52.9% [9/17]) remained hematuria. It suggested that proteinuria might be ameliorated, but hematuria remained, after intestinal or renal treatment. The outcome was similar to non-CD IgA nephropathy patients [41].

Limitations of this study: as a single-center retrospective study, this study requires further prospective long-term multicenter studies to observe the relationship between CD and kidney disease. Because of insufficient follow-up time, end-stage renal disease and mortality rate in Chinese patients with CD-associated renal disease are unknown, although previous reports found that the risk of end-stage renal disease in CD patients was about 5 times higher than that in non-CD patients regardless of age, sex, and comorbidities [42].

## Conclusions

The most common kidney biopsy diagnosis in CD is IgA nephropathy. Proteinuria might be in remission after intestinal or renal treatment, but hematuria remains. Therefore, proteinuria might play an important role in the assessment of the outcome of CD-related kidney disease.

## Data Availability

The data that supports the findings of this study is not publicly available since restrictions from the Sixth Affiliated Hospital of Sun Yat-sen University apply to the availability of these data. However, the data are available from the authors upon reasonable request and with permission of the Sixth Affiliated Hospital of Sun Yat-sen University.

## Abbreviations

IBD: Inflammatory bowel disease
CD: Crohn’s disease
eGFR: Estimated glomerular filtration rate
CKD-EPI: Chronic Kidney Disease Epidemiology Collaboration Formula
CDAI: Crohn’s disease activity index
CR: Complete remission
PR: Partial remission
R: Relapse
HE: Hematoxylin and eosin
PAS: Periodic acid-Schiff reagent
PASM: Periodic acid-silver methenamine
SD: Standard deviation
TBMN: Thin basement membrane nephropathy
GWAS: Genome-wide Association Study
5-ASA: 5-Aminosalicylic acid
